# Use of Continuous Glucose Monitoring in the Assessment and Management of Patients With Diabetes and Chronic Kidney Disease

**DOI:** 10.3389/fendo.2022.869899

**Published:** 2022-04-22

**Authors:** James Ling, Jack K. C. Ng, Juliana C. N. Chan, Elaine Chow

**Affiliations:** ^1^ Department of Medicine and Therapeutics, The Chinese University of Hong Kong, Prince of Wales Hospital, Hong Kong, Hong Kong SAR, China; ^2^ Hong Kong Institute of Diabetes and Obesity, The Chinese University of Hong Kong, Hong Kong, Hong Kong SAR, China; ^3^ Phase 1 Clinical Trial Centre, The Chinese University of Hong Kong, Prince of Wales Hospital, Hong Kong, Hong Kong SAR, China

**Keywords:** continuous glucose monitoring, end stage kidney disease (ESKD), dialysis, diabetes, type 2 (non-insulin-dependent) diabetes mellitus, diabetic kidney disease, diabetic nephropathy

## Abstract

In developed countries, diabetes is the leading cause of chronic kidney disease (CKD) and accounts for 50% of incidence of end stage kidney disease. Despite declining prevalence of micro- and macrovascular complications, there are rising trends in renal replacement therapy in diabetes. Optimal glycemic control may reduce risk of progression of CKD and related death. However, assessing glycemic control in patients with advanced CKD and on dialysis (G4-5) can be challenging. Laboratory biomarkers, such as glycated haemoglobin (HbA_1c_), may be biased by abnormalities in blood haemoglobin, use of iron therapy and erythropoiesis-stimulating agents and chronic inflammation due to uraemia. Similarly, glycated albumin and fructosamine may be biased by abnormal protein turnover. Patients with advanced CKD exhibited heterogeneity in glycemic control ranging from severe insulin resistance to ‘burnt-out’ beta-cell function. They also had high risk of hypoglycaemia due to reduced renal gluconeogenesis, frequent use of insulin and dysregulation of counterregulatory hormones. Continuous glucose monitoring (CGM) systems measure glucose in interstitial fluid every few minutes and provide an alternative and more reliable method of glycemic assessment, including asymptomatic hypoglycaemia and hyperglycaemic excursions. Recent international guidelines recommended use of CGM-derived Glucose Management Index (GMI) in patients with advanced CKD although data are scarce in this population. Using CGM, patients with CKD were found to experience marked glycemic fluctuations with hypoglycemia due to loss of glucose and insulin during haemodialysis (HD) followed by hyperglycemia in the post-HD period. On the other hand, during peritoneal dialysis, patients may experience glycemic excursions with influx of glucose from dialysate solutions. These undesirable glucose exposure and variability may accelerate decline of residual renal function. Although CGM may improve the quality of glycemic monitoring and control in populations with CKD, further studies are needed to confirm the accuracy, optimal mode and frequency of CGM as well as their cost-effectiveness and user-acceptability in patients with advanced CKD and dialysis.

## Introduction

Diabetic kidney disease (DKD) is now the leading cause of chronic kidney disease (CKD) and end-stage kidney disease (ESKD) in many countries. In 2014, DKD accounted for 50% of patients with ESKD in developed world (1). Data from the United States (US) suggested a slower decline in ESKD incidence compared with other diabetic complications including cardiovascular disease. The US Renal Registry reported a steady increase in incidence of ESKD due to diabetes up to 47% in 2017, compared with 15% in 1985 (2). In the Hong Kong Renal Registry, diabetes was the cause of ESKD in 50% of patients which had replaced glomerulonephritis as the leading cause of renal replacement therapy since 1998 (3). 

Patients with diabetes and CKD have increased risk of morbidity and premature mortality than those without renal complications. In the Hong Kong Diabetes Register, patients with CKD had 63% higher risk in all-cause mortality than their non-CKD counterparts, after adjusting for factors such as age, body mass index (BMI), blood pressure and use of oral glucose lowering drugs (OGLDs) (4). Patients with CKD had high risk of cardiovascular events which accounted for 40-50% of mortality in those with estimated glomerular filtration rate (eGFR) < 30 ml/min/1.73m^2^. This excess risk could not be explained by comorbid factors such as hypertension and dyslipidaemia (5) and might be attributed to additional factors such as vascular calcification, chronic inflammation and myocardial fibrosis (6). Patients with CKD are at increased risk and more vulnerable to hypoglyceamic episodes (4). In a cohort of over 30,000 US veterans with diabetes transitioning to dialysis, the frequency of hypoglycemia-related hospitalizations was associated with higher post-ESKD mortality in a dose-dependent manner (7).

Optimal glycemic control had been shown to delay progression of CKD and reduce death rate in diabetes. In the Diabetes Control and Complication Trial, 1441 patients with type 1 diabetes (T1D) were randomized to receive intensive or conventional insulin treatment. The risk of microalbuminuria was reduced by 34% in the intensive treatment group after at least four years of follow-up (8). The Action in Diabetes and Vascular Disease: Preterax and Diamicron MR Controlled Evaluation (ADVANCE) trial enrolled high risk patients with long duration of type 2 diabetes, (T2D), many of whom had prior history of complications. The in-trial reductions in the risk of ESKD was maintained during a total follow-up period of 9.9 years with a hazard ratio of 0.54 (29 events in the intensive treatment group and 53 events in the usual treatment group) (9). In a randomized controlled study of Japanese patients with 110 T2D lasting for 8 years, intensive insulin therapy reduced the rate of progression in nephropathy compared with conventional treatment (10). In the Dialysis Outcomes and Practice Pattern Study (DOPPS) including 9201 patients on dialysis with either T1D or T2D, there was a U-shaped relationship between HbA_1c_ and all-cause mortality. Using HbA_1c_ 7 – 8% as reference, there was 38% increased risk of mortality in patients with HbA_1c_ ≥9% and 21% for those with HbA1c <7% (11). Based on the available evidence, The Kidney Disease Improving Global Outcome (KDIGO) 2020 guideline recommended an optimal HbA_1c_ target range of 6.5-8.0% for patients with diabetes and CKD, with emphasis on individualization of targets taking age, comorbidities, life expectancy and hypoglycaemia risks into consideration (12).

Optimal glycemic management in patients with diabetes and CKD can be challenging, particularly in those with advanced CKD. Reasons include progressive decline in beta-cell function and increase in insulin resistance along with increased risk of severe hypoglycaemia and limited choices of OGLDs. Indeed, the heterogeneity in glycemic control amongst patients with CKD represents inter- and intra-individual variations amongst multiple interacting factors including insulin secretion, insulin resistance, renal clearance of insulin, renal gluconeogenesis and renal function. Increased insulin resistance in early CKD may be triggered by metabolic acidosis, uremic toxins, and chronic inflammation associated with reduced kidney function (13–16). With progression of CKD, the prolonged glucose-lowering effects of oral glucose lowering-drugs (OGLD) including insulin, together with reduced renal gluconeogenesis, shifts the balance towards increased risk of hypoglycaemia (17, 18). In patients with ESKD, around 30% had “burn-out diabetes” who required reduction or discontinuation of insulin treatment and OGLDs (18). In these patients, initiation of dialysis may remove uremic toxins with restoration of insulin sensitivity. Patients with “burnt-out diabetes’ often require only low-dose insulin treatment (19). On the other hand, the dialysis regimen and glucose content of dialysates can significantly influence day-to-day glucose profiles.

One of the greatest challenges in optimizing glycemic management is accurate assessment of glucose control. Conventional markers such as glycated haemoglobin (HbA_1c_),fructosamine or glycated albumin may be less reliable in in advanced CKD and ESKD. With the emergence of continuous glucose monitoring (CGM), this might be a helpful alternative in assessing and managing diabetes patients with advanced CKD and ESKD. The aim of this narrative review is to summarise current clinical evidence on the accuracy and utility of CGM in CKD patients. We have reviewed the literature on clinical reports, observational studies and clinical trials of use of CGM in CKD. Due to potential issues of sensor performance and the impact of dialysis regimens, we have devoted special attention to use of CGM in patients on haemodialysis and peritoneal dialysis, a challenging group who are prone to both hypoglycemic and hyperglycemic excursions.

## Challenges in Glycemic Assessment in CKD

The monitoring of glycemic status in patients with diabetes and CKD including ESKD is challenging. HbA_1c_, the gold standard as a laboratory glycemic marker, can be influenced by multiple factors in CKD. The formation of HbA_1c_ is dependent on the intensity and duration of non-enzymatic interaction between blood glucose and hemoglobin. At any one time, patients may have a mixture of erythrocytes with different ages and varying degrees of exposure to glucose. Therefore, agents that alter erythropoiesis and lifespan of red blood cells will affect HbA_1c_. For example, HbA_1c_ can be biased towards high values by iron or vitamin B12 deficiency due to reduced synthesis of red blood cells with increased relative amount of HbA_1c_. On the other hand, HbA_1c_ can be biased towards low values by iron therapy and use of erythropoietin stimulating agents (ESA) with increased turnover of red blood cells (20, 21). The uremic environment in patients with advanced CKD can stimulate carbamylation of haemoglobin which may interfere with HbA_1c_ assays using ion-exchange method, but this can be avoided by using other methods such as high-pressure liquid chromatography (22).

Alternative glycemic indicators such as glycated albumin (GA) and fructosamine have their own limitations in CKD. Extracellular GA is more susceptible to glycation than intracellular hemoglobin (23). Also, GA is unaffected by factors such as iron therapy and ESA frequently used in patients with CKD which can affect HbA_1c_ (21). Due to the shorter half-life of albumin, GA reflects recent glycemic control lasting for 2-3 weeks. However, GA can be affected by albumin metabolism. In patients with low albumin state or increased protein turnover due to chronic inflammation, GA can be falsely low or high (24). In patients treated with peritoneal dialysis (PD) with increased protein loss, GA value may underestimate true glycaemia (25). Although GA can be corrected for serum albumin to reflect the true distribution (26), GA can be affected by oxidative and uremic environments, as well as reduced renal clearance of advanced glycation end products, resulting in positive bias (27).

Fructosamine are ketoamines formed by glycation of albumin and other less abundant serum proteins (28). Although this biomarker involves a wider spectrum of glycated proteins, fructosamine suffers similar bias as GA due to abnormal albumin metabolism and increased protein loss in patients with CKD. In patients with diabetes without CKD and normal serum albumin level, increased albuminuria was associated with low fructosamine value. Besides, fructosamine is sensitive to the fluctuation of serum levels of immunoglobulins and low-molecular-weight molecules (29). In patients with CKD, the uremic environment with altered immunoglobulin levels may affect fructosamine levels (30).

## Overview of CGM

The introduction of continuous glucose monitoring (CGM) offers an alternative for more reliable and comprehensive glycemic evaluation in patients with CKD. Adherence to self-monitoring of blood glucose (SMBG) is often poor due to inconvenience of finger-pricking. In a survey conducted in China, only 40% of patients adhered to the recommended SMBG frequencies (31). Most commercially-available CGM devices are minimally-invasive by inserting a small filament into subcutaneous tissue for measurement of glucose in interstitial fluid. There is a dynamic equilibrium between interstitial glucose and blood glucose due to diffusion dependent on concentration gradient. The interstitial glucose is absorbed into the filament of the CGM device by capillary action. The concentration of interstitial glucose is determined by electrochemical reaction in the sensor (32). Minute-to-minute interstitial glucose readings are transmitted to and displayed in a mobile device, either a reader or smartphone app.

In general, CGM systems can be classified into three categories based on their principles of operation and clinical usage. For professional CGM devices, readings are principally used for glycemic assessment by health care professionals in clinical trial settings which may be blinded or unblinded to the user. Real-time CGM (rt-CGM) devices display readings to the user continuously and can incorporate hypoglycemic or hyperglycemic alerts and trend prediction. The intermittently-scanned or flash CGM devices display readings to user only when the user scans the transmitter (33). Real-time CGM and flash CGM are gaining popularity to facilitate self-monitoring in diabetes. In some countries, CGM devices are reimbursed or funded by public health systems for patients with T1D, including those on dialysis, and some patients with T2D receiving intensive insulin therapy (34).

## Performance of CGM Sensors in Advanced CKD and Dialysis

**Figure 1:**
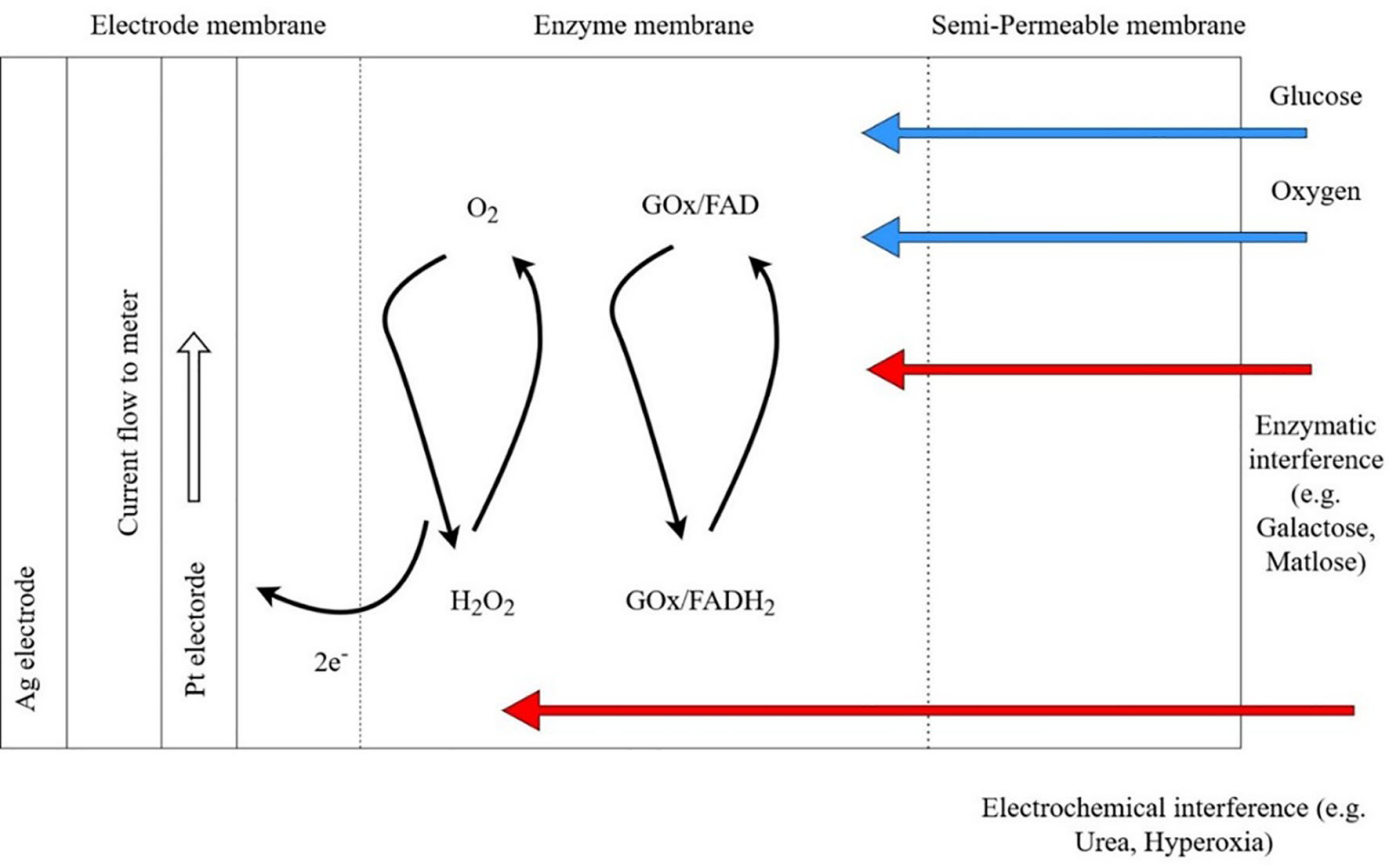
Potential enzymatic and electrochemical interference by substances commonly encountered in patients with chronic kidney disease using continuous glucose monitoring (CGM) systems. In the presence of oxygen (O_2_), energy in glucose (G) is gradually released in the form of electrons in a series of electrochemical chains catalyzed by Glucose oxidase (GO), an enzyme with Flavin Adenine Dinucleotide (FAD) as cofactor. Released electron is captured by the electrode membrane to generate electric current between platinum electrode and silver electrode. Platinum and silver are chosen for their excellent biocompatibility, electro-conductivity and non-toxicity. Blue arrows indicate normal substrate for electrochemical chain. Red arrows indicate potential interference of CGM sensors by for example galactose, maltose from peritoneal dialysis fluids. Enzymatic interference includes competitive inhibition on active site of GOx by inhibitors. Electrochemical interference includes interaction between the electrode and interfering chemicals that pass through the semi-permeable membrane. GOx, glucose oxidase; FAD, Flavin Adenine Dinucleotide; H_2_O_2_, hydrogen peroxide; Pt, platinum; Ag, Silver. Adapted from Boehm et al. (35).

The performance of CGM sensor is dependent on the enzymatic electrochemical reactions which may be subject to multiple interferences (**Figure 1**). In early CGM devices, interstitial glucose was detected by glucose oxidase-peroxidase method (36). This method continues to be used by some CGM systems due to the small size and rapid response time of the sensor. However, the electrodes often require pretreatment for attaching to the enzyme surface. Prolonged chemical reactions may pollute the surface of transducer and affect the electrochemical response (37). Both endogenous and exogenous substances may cause interference of the electrochemical sensing of the oxidase-peroxidase reaction.

In patients with advanced CKD, hypoxia or hyperoxia can give rise to false sensor glucose values by changing the oxygen concentration at the initiation of the glucose oxidase chain reaction (38). There had been reports on the effects of hematocrit in altering glucose readings of glucometers that use glucose-dehydrogenase or glucose-oxidase methods (39). Endogenous substances such as uric acid and uremia may affect sensor performance. Ogawa et al. demonstrated significant interference of uric acid, a reducing agent, on glucometers using glucose oxidase method comparing with laboratory glucose hexokinase reference (40) However, uric acid did not significantly interfere with sensor performance of a microdialysis-based CGM system (41). There are no dedicated studies evaluating the effect of pH on CGM sensor performance in ESKD. In critically ill patients, extreme pH <6.95 may affect the performance of point-of-care glucometers but not within pH range 6.97-7.84 (42). One study evaluated the effect of pH on the accuracy of CGM in a group pediatric intensive care patients and did not observe any significant effect (43). It is unknown whether fluid status might affect CGM performance in CKD patients due to lack of dedicated studies, however, a small study comparing hospitalized diabetes patients with and without congestive heart failure shown no differences in sensor accuracy (44).

Amongst exogenous substances, ascorbic acid, paracetamol, xylose, and ethanol have the potential to interfere with glucose oxidase sensors (45, 46) Other metabolites of icodextrin, such as maltose, also interfere with glucose dehydrogenase-based detectors using pyrroloquinoline quinone (GDH-PQQ) due to lack of selectivity on glucose (47). Use of GDH-PQQ glucometers can result in falsely elevated glucose readings in patients with PD using icodextrin dialysate. On the other hand, glucose-oxidase based capillary blood glucometers are mostly unaffected by icodextrin (35). Most commercially available CGM system use glucose-oxidase sensors although interference of CGM sensors by icodextrin had not been explored.

Performance of commercially available enzyme-based CGM systems have been validated in small numbers of patients on dialysis. For example, Yajima et al. evaluated accuracy of two CGM systems, the Freestyle Libre Pro and Medtronic iPro2™ with Enlite™ sensor versus capillary blood glucose in patients undergoing HD. For Freestyle Libre, 49% of readings fell within the Parkes Error Grid zone A and 51% in zone B. The Medtronic Ipro2™ sensor exhibited smaller deviations with 93% of readings within zone A and 6.3% in zone B which are regarded as clinically acceptable. Mean absolute relative difference (MARD) was 19.5% ± 13.2% for Freestyle Libre versus 8.1% ± 7.6% for Medtronic iPro2 (48). In a three-week study comparing the accuracy Freestyle Libre versus capillary blood glucose in 12 patients on haemodialysis, the MARD was found to be higher than people without ESKD (49). Only one study had evaluated the accuracy of Medtronic iPro2™ with Enlite™ sensor in 40 patients on PD. When compared with capillary blood glucose, MARD was 14%-19% (50). The accuracy of Dexcom sensors in haemodialysis is being investigated in ongoing trials (NCT04217161). Larger evaluation studies of sensor glucose against values measured by standard laboratory analyzers are needed in patients on different dialysis regimens.

## Use of CGM Metrics in Glycemic Assessment in CKD

Several studies analyzed the correlation between HbA_1c_, fructosamine, GA and average sensor glucose across different CKD stages (**Table 2**). In general, correlation between HbA_1c_ and mean sensor glucose values tend to fall in CKD stage G4-5, in part confounded by differences in use of iron and ESA and blood haemoglobin. Lo and colleagues reported good correlation of mean CGM-glucose with HbA_1c_ (*r*= 0.79) in patients with eGFR 30-59 ml/min/1.73m^2^ but fell (*r*=0.34) in participants (n=43) with eGFR below 30 ml/min/1.73m^2^ (51). In another study involving 25 patients with diabetes, the authors reported weak correlation (*r*=0.38) between mean CGM-glucose and HbA_1c_ in patients with eGFR <30ml/min/1.73m^2^ (52).

Nathan et al. first estimated HbA_1c_ by linearly regressing mean sensor glucose with HbA_1c_ in intensively-treated patients with T1D in the Diabetes Control and Complication Trial (DCCT) (53). Bergenstal et al. later proposed the use of glucose management index (GMI) to reflect the relationship between CGM glucose and HbA_1c_ (54). However, these equations were derived predominantly from T1D and T2D patients with normal renal function and the reliability of the current GMI equation is unknown in patients with CKD (55). In one cohort, Zelnick and colleagues reported similar correlations between GMI and HbA_1c_ of 0.78 in patients with eGFR >30 ml/min/1.73m^2^ (n=80) and 0.76 in those with <30 ml/min/1.73m^2^ (n=24) (56). Nevertheless, the 2020 KDIGO guideline suggested GMI might be an alternative index for guiding treatment in patients with CKD G4-5 or dialysis where HbA_1c_ had been shown to be less reliable (12). (**Table 1**).

**Table 1:** KDIGO 2020 recommendations on assessment of glycaemia in patients in chronic kidney disease (CKD) stages 1-4 (12).

Population	HbA_1c_			Glucose management indicator
	Measure	Frequency	Reliability	
CKD G1-G3b	Yes	Twice per year Up to 4 times per year if not achieving target or change in therapy	High	Occasionally useful
CKD G4-G5 Including treatment by dialysis or kidney transplant	Yes	Twice per year Up to 4 times per year if not achieving target or change in therapy	Low	Likely useful

**Table 2:** Summary of studies assessing correlation between continuous glucose monitoring (CGM) metrics and glycemic markers in patients with chronic kidney disease (CKD).

Study	Year	n	Subjects on ESA	Mean blood haemoglobin (g/dL)	CGM metric	Laboratory marker	Reported correlation
Frederick et al. (52)	2012	50	Yes	no CKD: 14.3± 1.1 G4 & G5: 11.5 ± 1.5	Mean sensor glucose	HbA_1c_	No CKD: n= 25, *r*= 0.66 G4 & G5: n= 25, *r*= 0.38
Lo et al. (58)	2014	147	Yes	no CKD: NA G3b: 12.3 ± 1.1 G4: 11.4 ± 1.6 G5: 11.7 ± 1.0	Arithmetic mean CGM-SMBG glucose	HbA_1c_	No CKD: n= 104, *r*=0.74 G3b: n= 14, *r*= 0.79 G4 & G5: n= 29, *r*= 0.34
Lubaina et al. (59)	2019	80 (with 49 G4-G5)	Yes	NA	Mean sensor glucose	HbA_1c_	G3b: n= 31, *r*= 0.85 G4 & G5: n= 49, *r*= 0.81
					Mean sensor glucose	Fructosamine	G3b: n= 31, *r*= 0.69 G4 & G5: n= 49, *r*= 0.51
Zelnick et al. (56)	2020	104 (with 22 G4-G5)	No	no CKD: 13.1 ± 2.0 CKD: 12.2 ± 1.6	GMI	HbA_1c_	No CKD: n= 24, *r*= 0.76 CKD (G3b-G5): n= 80, *r*= 0.78
					GMI	Fructosamine	No CKD: n= 24, *r*= 0.72 CKD (G3b-G5): n= 80, *r*= 0.78
					GMI	Glycated albumin	No CKD: n= 24, *r*= 0.63 CKD (G3b-G5): n= 80, *r*= 0.71

ESA, Erythropoietin stimulating agent; GMI, glucose management indicator; HbA_1c_, glycated haemoglobin; SMBG, self-monitoring of blood glucose; NA, not available.

Of equal if not greater importance is the use of time-in-ranges which describes the proportion of time the patient spent in hyperglycemia or hypoglycaemia range. In 2019, at the Advanced Technology and Treatment for Diabetes (ATTD) Conference, there was consensus on using a series of CGM-derived metrics as clinical targets for glycemic management. The recommended target in an adult patient with T2D and without complications was >70% Time in range (TIR, % time sensor glucose >3.9 and <10 mmol/L), <25% time in Time above range reflecting significant hyperglycemia (TAR, % time sensor glucose >10 mmol/L), <5% time below target suggesting hypoglycaemia (TBR, % time sensor glucose <3.9 mmol/L) with a Coefficient of Variation < 36% (%CV = SD (standard deviation) of sensor glucose/mean sensor glucose) (57). However, the validity of TIR targets and the prognostic values of CGM-derived metrics on complications and death need to be confirmed in clinical trials involving patients with advanced CKD and dialysis (12).

## Glycemic Profiles of Patients on Dialysis

CGM systems provide comprehensive 24-hour profiles for assessment of relationships between glycemic variation, timing of dialysis regimens and insulin administration. In addition to the aforementioned CGM metrics, most CGM systems now provide standardized ambulatory glucose profiles (AGPs) which provide a graphical representation of 24-hour sensor glucose trends. **Table 3** summarizes evaluation studies of CGM in patients on HD or PD.

**Table 3:** Key Continuous Glucose Monitoring (CGM) studies in patients on hemodialysis or peritoneal dialysis.

Study	Year	CGM device; study duration	Mode of dialysis	Participants	Key findings
Kazempour-Ardebili et al. (60)	2009	Unknown (48 hours)	HD	19 T2D	Mean sensor glucose was lower during HD days than HD-free days Mean sensor glucose and sensor glucose AUC on post-HD days were significantly higher than HD days Nocturnal sensor mean glucose and sensor glucose AUC showed same pattern
Gai et al. (51)	2014	Medtronic Ipro2 (6 Days, Blinded)	HD	12 DM	Median CGM reading was lower than dialysate glucose concentration for 87% of time Post-HD hyperglycemia observed in 75% of subjects
Jung et al. (61)	2010	Medtronic Gold (3 days, Blinded)	HD	9 T2D	Significantly lower mean sensor glucose during HD sessions regardless of glucose concentration of dialysate solution Hypoglycaemic events were concentrated on the day of HD session
Jin et al. (62)	2014	Medtronic Minimed (3 days, Blinded)	HD	36 T2D, 10 non-DM	Significantly lower mean sensor glucose during HD sessions compared with peri-HD sessions in patients with or without diabetes Diabetes patients suffered greater loss in glucose during HD session, and greater post-HD hyperglycemia than their non-diabetes counterparts
Mirani et al. (63)	2010	GlucoDay (2 days, Blinded)	HD	12T2D	Hypoglycaemia observed in post-HD period Rebounded hyperglycemia observed after post-HD hypoglycaemia Significant higher glycemic variability in SD for HD day when compared with non-HD day
Padmanabhan et al. (64)	2018	Freestyle LibrePro (14 days, Blinded)	HD	16 DM + 16 non-DM	Significantly fewer hypoglycaemic episodes during days of dialysis with glucose-rich dialysate than glucose-free dialysate Significantly lower % TBR and lower % TAR during days of dialysis with glucose-rich dialysate than glucose-free dialysate Significantly less loss in effluent glucose irrespective to diabetic state during days using glucose-rich dialysate than glucose-free dialysate
Hayashi et al. (65)	2021	Medtronic Gold (2 days, blinded) & Medtronic Ipro 2 (2 days, blinded)	HD	98 T2D	Reduced sensor glucose irrespective of the dialysate glucose concentration (100, 125, 150 mg/dl) 50% of patients reached a nadir lower than dialysate glucose concentration, 21% of patients developed asymptomatic hypoglycaemic events during HD and post-HD session Glycemic variability and % TBR increase in patients who experienced hypoglycaemic events than their counterparts without events
Schwing et al. (66)	2004	Medtronic Minimed (3 Days, Blinded)	PD	7 DM	Increase in sensor glucose after dialysate exchange in two representative patients
Lee et al. (67)	2013	Medtronic Minimed (3 days, Blinded)	PD	25 DM	Increase in sensor glucose within 60 minutes of refilling glucose-rich dialysate Reduced sensor glucose in icodextrin dialysate after refilling
Marshall et al. (68)	2003	Medtronic Minimed (3 days, Blinded)	PD	8 DM	Mean sensor glucose and glycemic variability in % CV significantly lower when switching from glucose-rich dialysate to glucose-free dialysate
Qayyum et al. (69)	2016	Dexcom G4 (7 days, real time CGM)	PD	60 T1/T2D	Sensor-detected hypoglycaemia in subgroup of patients with A1c >9%
Okada et al. (70)	2015	Medtronic Gold (3 days, Blinded)	PD	20 DM	Frequent sensor-detected hyperglycemia observed despite well controlled A1c
Skubala et al. (71)	2010	Medtronic Minimed (3 days, Blinded)	PD	16 T1/T2D 14 non-DM 13 healthy control	Significant difference in mean sensor glucose and mean changes in sensor glucose after dialysate exchange in subgroup of patients with HPT versus H-APT Peritoneal transport status influenced mean 24-hour sensor glucose in non-diabetic patients on PD as well as mean sensor glucose and mean changes in sensor glucose after dialysate exchange in diabetic patients on PD

AUC, area under the curve; HD, hemodialysis; PD, peritoneal dialysis; HPT, high peritoneal transport; HAPT, high average peritoneal transport; T1D, Type 1 diabetes; T2D, Type 2 diabetes; DM, diabetes mellitus; TBR, time below range; TAR, time above range.

## Glycemic Profiles During Hemodialysis (HD)

**Figure 2:**
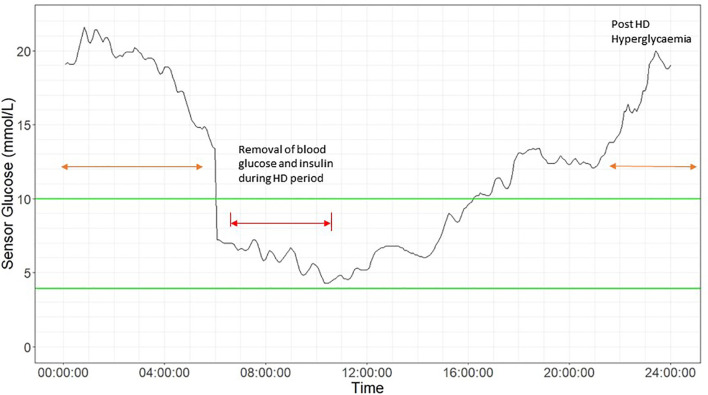
Glycemic disarray showing marked variability in patients during haemodialysis (HD) and post-HD period. 24-hour CGM glucose profile in a 58-year-old man with type 2 diabetes on HD using glucose- free dialysate. He was treated with insulin glargine 24 units in the morning and alogliptin 6.25mg daily with HbA_1c_ of 8.2%. The HD period is indicated by red arrow, showing an acute drop in sensor glucose, followed by post HD-associated hyperglycemia (orange arrow) up to 20 mmol/l at midnight. Green lines indicates target range (3.9 mmol/L to 10 mmol/L).

In patients on HD, the composition of the dialysate and dialysis membrane both contribute to glycemic variability during HD and in the post-HD period. Differences in glucose profiles have also been reported between HD and non-HD days. The phenomenon of “glycemic disarray” in HD has been described, referring to the fall in glucose during HD followed by rebound hyperglycemia in the post-HD period (**Figure 2** **)**. HD-induced hypoglycaemia is frequently observed. In early studies, Takahashi et al. demonstrated reduction in plasma glucose concentration from pre-dialyser site to post-dialyser site in patients under a dialysate of 5.55 mmol/L glucose. This reduction in serum glucose within the dialyzer might be induced by dialysate-stress triggered diffusion of plasma glucose into erythrocyte, as well as loss into the dialysate (72). In general, patients might lose around 15-30 g of glucose during HD session. In patients with ESKD, defective counter-regulatory effects, reduced renal gluconeogenesis and hypoglycaemia unawareness might result in frequent asymptomatic hypoglycaemic events. In 17 patients with T2D on HD, the mean sensor glucose was lower during the on-dialysis than the off-dialysis days (60). In 12 patients on dialysis, Gai et al. reported that the median CGM glucose level was below the concentration of dialysate of 5.55 mmol/L during most of the HD session (87% of time) (51). In 9 patients with T2D, Jung et al. reported significant reduction in mean sensor glucose during HD session, regardless of the glucose concentration of dialysate solution (5.55 – 11.1 mmol/L) with most of the hypoglycaemic events occurring on the day of HD (61). In 46 patients with ESKD with or without diabetes, Jin et al. reported a significant reduction in mean sensor glucose during HD session irrespective of the status of diabetes although patients with diabetes had greater glucose loss during HD session (62).

In a recent study involving 98 Japanese patients with T2D on HD who had 2-day CGM, sensor glucose showed a sustained decline irrespective of dialysate glucose concentration with 50% of patients with diabetes reaching a glucose nadir lower than the dialysate concentration. In the whole group, 21% experienced HD-related hypoglycaemia <3.9 mmol/L either during the HD session or post-HD and before the next meal. There were no difference in terms of clinical characteristics (e.g. body mass index, duration of diabetes, insulin treatment) and traditional glycemic markers (e.g. HbA_1c_ and GA), between patients with HD-related or post-HD hypoglycaemia and patients without hypoglycaemia. Despite an average HbA_1c_: 6.4% ± 1.2% for these T2D patients, asymptomatic HD-related hypoglycaemia was frequent and the HD-related hypoglycaemia was only captured by CGM (65).

Rebound hyperglycemia during the post-HD period may be related to choices of dialysate and dialysis membrane, which can influence plasma insulin concentrations during dialysis (73, 74). Insulin is readily removed from plasma by diffusion owing to its small molecular size and low protein-binding capacity. However, during HD, most of the insulin is removed *via* adsorption with dialysis membrane through electrostatic and hydrophobic interactions resulting in hyperglycemia in the post-HD period. The clearance of insulin by absorption depends on the type of dialysis membrane, with greatest absorption in polysulfone membrane and lowest absorption in polyester-polymer alloy (19). The counter-regulatory hormonal responses to HD-induced hypoglycaemia could increase insulin resistance and trigger post-HD hyperglycemia. Kazempour-Ardebili et al. demonstrated that nocturnal sensor glucose was significantly higher on the HD-day than HD-free day (60). This was also confirmed by other studies where time of HD-session was reported in the 24-hour CGM profile (51, 61, 63). Jin et al. confirmed post-HD hyperglycemia especially in patients with diabetes compared with their non-diabetic counterparts (62). Padmanabhan et al. evaluated the effects of different dialysate and dialysis membranes on glycemic control. In a study of 38 patients with and without diabetes, HD-induced hypoglycaemia and post-HD hyperglycemia occurred with the use of glucose-free dialysate but the fluctuation could be attenuated by using glucose-containing dialysate (64). Both HD-induced hypoglycaemia and post-HD hyperglycemia may contribute to heightened glycemic variability, increased oxidative stress and inflammation with worsening of clinical outcomes. By using CGM, these silent events may be detected early to inform treatment.

## Glucose Profiles During Peritoneal Dialysis (PD)

**Figure 3:**
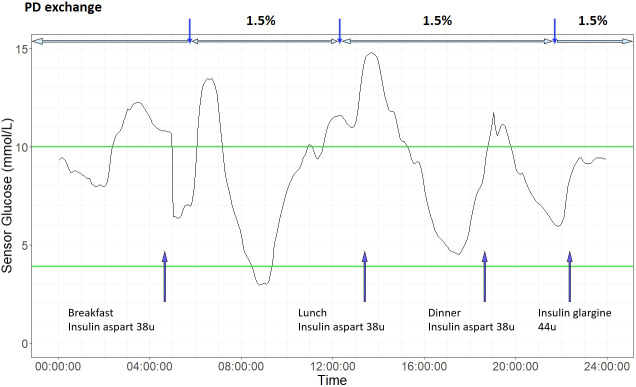
An illustrative 24-hour ambulatory glucose profile in a patient with type 2 diabetes on continuous ambulatory peritoneal dialysis (CAPD). He is on three 1.5% dextrose exchanges daily and basal-bolus insulin regimen. Laboratory measures of glycemic control were HbA_1c_ 7.5% and Fructosamine 224 µmol/L. Based on CGM metrics, glucose management indicator (GMI) was 6.9%, coefficient variation (CV) 36.9%. Blue arrow on bottom indicates times of insulin injection and meal intake. Vertical blue arrow on top indicate PD exchange timing, and horizontal blue arrow on top indicate PD exchange period. Green lines indicates target CGM glucose range (3.9 mmol/L to 10 mmol/L).

One of the determining factors of glycemic profile in patients with PD is the rate of peritoneal absorption of glucose, which is in turn affected by glucose concentration of dialysate, dwell time, and status of membrane transport (75). Ultrafiltration by peritoneal membrane is created by either crystalloid osmosis using a higher glucose concentration in the dialysate, or by colloid osmosis using large colloid agents like icodextrin (76). Icodextrin solution contains a mixture of glucose polymers which are slowly absorbed *via* lymphatics. Together with its osmotic effect, icodextrin leads to sustained ultrafiltration and is widely used as an alternative osmotic agent to dextrose especially in dialysate with long dwelling time (77). Early observational studies using CGM showed that patients with PD spent a large proportion of time in hyperglycemia (66). In a study of 20 patients with well-controlled T1D and T2D and mean HbA_1c_ of 5.9% who were dialysed on glucose-containing dialysates, patients spent on average 33% time above 10 mmol/l and 1% time below 3.9 mmol/l (70). Lee et al. evaluated the impact of glucose influx from dialysate in 25 patients with diabetes on maintenance PD. In patients using glucose-based dialysate, the sensor glucose levels increased by 7-8 mg/dL within 1 hour of exchange using glucose-containing dialysate. The glycemic excursion was similar with 1.25% and 2.25% glucose solutions with larger increments observed with 3.86% glucose solutions (67). **Figure 3** shows an example of CGM profile in a patient on continuous ambulatory peritoneal dialysis (CAPD).

Icodextrin is associated with stable or even decreases in CGM sensor glucose during PD dwells (67). Marshall et al. demonstrated the effect of switching dialysate on CGM profiles in 8 patients with PD. Switching from three 1.36% glucose exchanges and one 3.86% glucose exchange to two bags of 1.36% glucose exchange, one bag of amino acid exchange and one bag of icodextrin was associated with lower sensor glucose and glycemic variability (68). In a retrospective study of 60 patients with 95% of them receiving icodextrin dialysate, the CGM-detected time in hypoglycaemia was 5% which was often asymptomatic (69).

The diffusing capacity of the peritoneal membrane is another crucial factor in determining glycaemia. The exchange rate of serum-dialysate glucose is dependent on the osmotic pressure, as well as the transport status of peritoneal membrane. Osmotic gradient between dialysate and peritoneum is rapidly lost in patients with high transporter status due to rapid absorption of glucose from dialysate (78). As a result, these patients might have high risk of PD-related hyperglycemia. Skubala et al. demonstrated the effect of peritoneal transport status using CGM in 30 patients with and without diabetes. In their study, patients on 1.36% and 2.27% glucose dialysates had similar HbA_1c_, mean 24-hour CGM-glucose, mean post-PD glucose, and mean post-PD increment in glucose. However, mean post-PD glucose and mean post-PD increment in glucose was significantly different in patients with high peritoneal transport (HPT) and high average peritoneal transport (HAPT), even in nondiabetic individuals (71).

Another modifiable factor of CGM-glucose is the timing, route and dose of insulin administration in patients on PD. Subcutaneous basal bolus insulin regimen are effective regimens in patients with T1D or T2D on PD but require frequent self-monitoring (50). Intraperitoneal (IP) delivery of insulin can counteract the glucose absorption from dialysate. However, there are no standardised recommendations on initiation or titration of IP insulin for different dialysates (79). Dose adjustments are often based on infrequent fasting and post-meal capillary blood glucose with CGM having the potential to guide adjustment of insulin therapy in patients on PD.

In summary, patients with diabetes on HD or PD display distinct glycemic profiles and patterns which can be comprehensively assessed by CGM. Apart from patient factors (e.g. beta-cell function, PD transporter status), there are a number of modifiable treatment factors, such as choices of dialysate, dialysis regimen and doses/timing of insulin, where data from CGM can help optimize treatment.

## Use of Personal CGM PatientsIn Advanced CKD or Maintenance Dialysis

Personal use of real-time (rt) or flash CGM devices may reduce hypoglycaemia and improve glycemic control in patients with diabetes without CKD. The benefits of CGM use in patients with T1D on improving glycemic control are now well-established. In the Randomized Controlled Trial Examining the Benefit of CGM Use for Adults with T1D on Insulin Injections (DIAMOND) trial (80), there was a significant HbA_1c_ difference of -0.6% in favour of rt-CGM versus standard SMBG after 24 weeks of intervention in T1D patients on multiple daily injection (MDI). In another randomized study involving 161 patients with T1D treated with MDI, a similar significant difference of -0.43% in HbA_1c_ in favor of rt-CGM versus standard SMBG was reported after 26-weeks of intervention and during 17-weeks of post-intervention washout period (80, 81). In an open-labelled randomized trial in adults with well-controlled T1D on MDI (REPLACE-BG trial), use of flash CGM without confirmatory SMBG was safe and reduced hypoglycaemia (82) with improved treatment satisfaction (83).

Several pilot and small-scale studies supported the potential beneficial effects of professional CGM in patients on HD or PD, whilst data on continuous personal use was limited. Most studies explored the use of blinded CGM for treatment titration. In a pilot-study, Képénékian al. used blinded CGM in 28 T2D patients on HD with suboptimal glycemic control for 54 hours at baseline and during a 3-month follow-up period. After 3 months of intervention, the CGM-adapted insulin regimen was associated with greater reduction in HbA_1c_ without increasing symptomatic hypoglycaemia (84). The DIALYDIAB pilot study involved 15 patients with T1D or T2D and compared the effect of blinded-CGM SMBG using a two-period design. Use of blinded CGM triggered more frequent treatment adjustments compared with SMBG alone. This resulted in better glucose profile with significantly lower HbA_1c_ and time above range without increasing hypoglycaemic episodes (85). There are also few studies in patients with PD where CGM was used to assess effects of structured education (50) or compare different glucose lowering drug regimens (86). These studies demonstrated the potential of CGM in promoting patient self-management and informing providers in treatment adjustment to improve glycemic control.

CGM systems have the potential to be combined with automated insulin delivery in closed-loop systems, also referred to as an ‘artificial pancreas”. A recent randomized trial evaluated a fully automated closed-loop system against standard insulin therapy in 26 patients with T2D on HD using a cross-over design (87). In this study, TIR was significantly higher (57.1% *versus* 42.5%) and time above and below range were lower (TAR: 42.6% *versus* 56.6%, TBR: 0.12% *versus* 0.17%) in the closed-loop phase. The mean sensor glucose was also significantly lower in the closed-loop *versus* control phase (10.1 mmol/L *versus* 11.6 mmol/L). Of note, the time spent in extreme hyperglycemia (defined as >20 mmol/L) was significantly lower during the closed-loop phase than the control phase (1.8% *versus* 6.7%). However, the system was given only for short-term use operated by healthcare professionals in a clinic setting rather than home use.

The ease of operation of personal CGM systems in patients with ESKD with multiple comorbidities need to be considered. Many patients with ESKD may have visual impairment due to retinopathy or cataracts, skin problems and cognitive issues that limit their ability to operate these devices. However, personal CGM with real-time alerts might benefit patients with ESKD on complex insulin regimens or vulnerability to hypoglycemia. Future research is required to investigate the utility and cost-effectiveness of personal CGM in patients with advanced CKD and dialysis.

There are some limitations in the use of CGM for patients under dialysis. Apart from potential sensor interference from endogenous and exogenous substances (46), accuracy of CGM is lower in the hypoglycemic range and under rapid changes in blood glucose values (88–90). False hypoglycaemic alerts may occur more frequently under these conditions, which may lead to unnecessary treatment. A confirmatory SMBG value is advisable for treatment decisions at these extreme glucose values. Additionally, repeated false positive alerts could lead to alarm fatigue and increase patient anxiety.

## Conclusions

Optimal glycemic control will delay progression of CKD and improve clinical outcomes. HbA_1c_ and alternative glycemic markers have limitations particularly in patients with advanced CKD. With the advent of CGM, it is now possible to monitor the glycemic status with better precision in patients with CKD. Professional CGM can inform health care professionals on glucose profiles not provided by HbA_1c_ in patients with CKD to optimize treatment regimens. Real-time or flash CGM provide instant or timely feedback to users on impact of meals and treatment on glucose excursion. The inclusion of real-time alerts in CGM, displayed in smart devices, can provide early warnings against hyperglycemia and hypoglycaemia. This information may improve the safety of prescription of GLDs and insulin in these high-risk patients. Finally, the integration of these CGM system with fully automated closed loop insulin delivery systems offer the potential of more precise control.

The use of CGM in patients with ESKD has revealed distinct glycemic patterns during maintenance dialysis. Glycemic pattern in patients under HD are impacted by the glucose concentrations in dialysate and choices of dialysis membrane. Glucose-free dialysate is generally preferred due to lower cost and chance of bacterial infection. However, glucose-containing dialysate may reduce HD-related hypoglycaemia and post-HD hyperglycemia, especially in patients with diabetes. Health care professionals should consider providing glucose-containing dialysate, replenishing post-HD glucose loss by snacks, or adjusting insulin regimen to avoid HD-related glycemic excursion. Similar to HD, glycemic patterns in PD patients are impacted by diasylate glucose concentration and peritoneal membrane transport state. Health care professionals should consider glucose influx from glucose-rich dialysate and adjust insulin treatment to maintain a stable blood glucose. Although switch to glucose-free dialysates may theoretically reduce glucose influx, randomized trials suggested this might be associated with adverse outcomes (91). Pending further evidence, a careful adjustment of insulin and dialysate regimens in patients under PD may strike the balance between optimizing glycemic control and ultrafiltration. Future studies using CGM should be conducted to investigate whether the use of personal CGM with glycemic alerts will reduce hypoglycaemia and complications and improve long-term outcomes in patients with advanced CKD and dialysis.

## Author Contributions

JL and EC conceived the idea of the paper. JL researched and wrote the manuscript, JL, JN, EC, and JC critically revised the manuscript. All authors contributed to and approved the final manuscript.

## Conflict of Interest

EC has received institutional research support form Medtronic Diabetes and Powder Pharmaceuticals Inc.

The remaining authors declare that the research was conducted in the absence of any commercial or financial relationships that could be construed as a potential conflict of interest.

## Publisher’s Note

All claims expressed in this article are solely those of the authors and do not necessarily represent those of their affiliated organizations, or those of the publisher, the editors and the reviewers. Any product that may be evaluated in this article, or claim that may be made by its manufacturer, is not guaranteed or endorsed by the publisher.

## Abbreviations

CKD, Chronic Kidney Disease; ESKD, End-Stage Kidney Disease; OGLD, Oral Glucose Lowering Drugs; eGFR, estimated Glomerulus Filtration Rate; KDIGO, Kidney Disease Improving Global Outcome; CGM, Continuous Glucose Monitoring; SMBG, Self-Monitoring Blood Glucose; PD, Peritoneal dialysis; HD, Haemodialysis; TAR, Target Above Range; TIR, Target In Range; TBR, Target Below Range; CV, Coefficient of Variation.
